# A Drug Carrier for Sustained Zero-Order Release of Peptide Therapeutics

**DOI:** 10.1038/s41598-017-05898-6

**Published:** 2017-07-17

**Authors:** Ya-Nan Zhao, Xiaoyu Xu, Na Wen, Rui Song, Qingbin Meng, Ying Guan, Siqi Cheng, Danni Cao, Yansheng Dong, Jiankun Qie, Keliang Liu, Yongjun Zhang

**Affiliations:** 1 State Key Laboratory of Medicinal Chemical Biology and Key Laboratory of Functional Polymer Materials, Institute of Polymer Chemistry, College of Chemistry, Nankai University, and Collaborative Innovation Center of Chemical Science and Engineering (Tianjin), Tianjin, 300071 China; 2 State Key Laboratory of Toxicology and Medical Countermeasures, Beijing Institute of Pharmacology and Toxicology, Beijing, 100850 China

## Abstract

Peptides have great potential as therapeutic agents, however, their clinic applications are severely hampered by their instability and short circulation half-life. Zero-order release carriers could not only extend the circulation lifetime of peptides, but also maintain the plasma drug level constant, and thus maximize their therapeutic efficacy and minimize their toxic effect. Here using PEGylated salmon calcitonin (PEG-sCT)/tannic acid (TA) film as an example, we demonstrated that hydrogen-bonded layer-by-layer films of a PEGylated peptide and a polyphenol could be a platform for zero-order peptide release. The films were fabricated under mild conditions. The second component, TA, is a natural product and presents potential therapeutic activities itself. Unlike common carriers, the new carrier releases the peptide via gradual disintegration of the film because of its dynamic nature. The release of PEG-sCT follows a perfect zero-order kinetics without initial burst release. In addition the release rate could be tuned via external stimuli, such as pH and temperature. When implanted in rats, the films could remain the plasma level of PEG-sCT constant over an extended period. Accordingly, the serum calcium level was reduced and maintained constant over the same period, suggesting an improved therapeutic efficacy of the released drug.

## Introduction

The tremendous advances in biotechnology are introducing more and more peptide therapeutics for the treatment of various diseases, particularly metabolic diseases and cancers^1–3^. Unfortunately, their intrinsic weaknesses, e.g. poor chemical and physical stability and therefore a short circulating plasma half-life, severely limited their clinical applications^4^. One method to extend circulation lifetimes is via PEGylation^5^. It was well documented that PEG conjugation could protect the fragile drug from enzymatic digestion, slow its filtration by the kidneys, and thus increase its retention in the circulation^6^. Another important approach is the using of drug carrier. Encapsulation of peptide therapeutic in a carrier could not only protect the drug against degradation but also allow its sustained release, leading to prolonged systemic exposure to the drug. Up to now, many carriers were designed for peptide release, including biodegradable microparticles (particularly poly(D,L-lactide-co-glycolide) (PLGA) microparticles)^7, 8^, micelles^9^, and hydrogels^10^. PEG conjugations can be regarded as a type of drug carriers too, in which the drug is chemically bound, instead of physically encapsulated^11^.

Although significant progress has been achieved in the development of drug carriers for peptide release, some challenges still remain. Particularly the carriers usually release the drug in a “fast-then-slow” manner. The rapid release at the initial stage will result in a high plasma drug level, which will pose a serious toxicity threat for the patients^8^. For example, biodegradable microspheres, which were extensively investigated for peptide release, usually suffer from a significant initial burst release^8, 12^. Initial burst was also observed in peptide releasing hydrogels both *in vitro* and *in vivo*, and is considered a limiting factor for their applications^10^. At the late stage of release, however, as the release rate slows down gradually, the plasma drug level will finally drop to a level not high enough to achieve therapeutic effects^13^. As a result, only a small portion of the precious drug will exert its therapeutic effects.

In this context, an ideal carrier should allow for zero-order release, i.e., the drug release rate is constant over the entire release duration^14, 15^. In this way, the fluctuation of the plasma drug level will be minimized. The plasma drug level will be maintained within the therapeutic window throughout the entire release period, leading to the highest therapeutic efficacy. Despite of intensive efforts in the last decades, however, achieving zero-order release still remains a big challenge^16^. Although many carriers and devices were designed for zero-order drug release, many of them are complex, expensive, and their manufacture is difficult and time consuming^15^. New methods for zero-order release are still highly desirable^15–26^. Here we designed a new carrier which allows for zero-order release of peptide drugs. Initial burst release is completely avoided. Preliminary *in vivo* tests suggest this carrier could maintain the plasma drug level constant over an extended period and thus achieve therapeutic effects for a long time. It is noteworthy that peptides can not only be used as therapeutics, but also widely used to construct drug carriers for drug delivery^27–30^.

## Results

**Figure 1:**
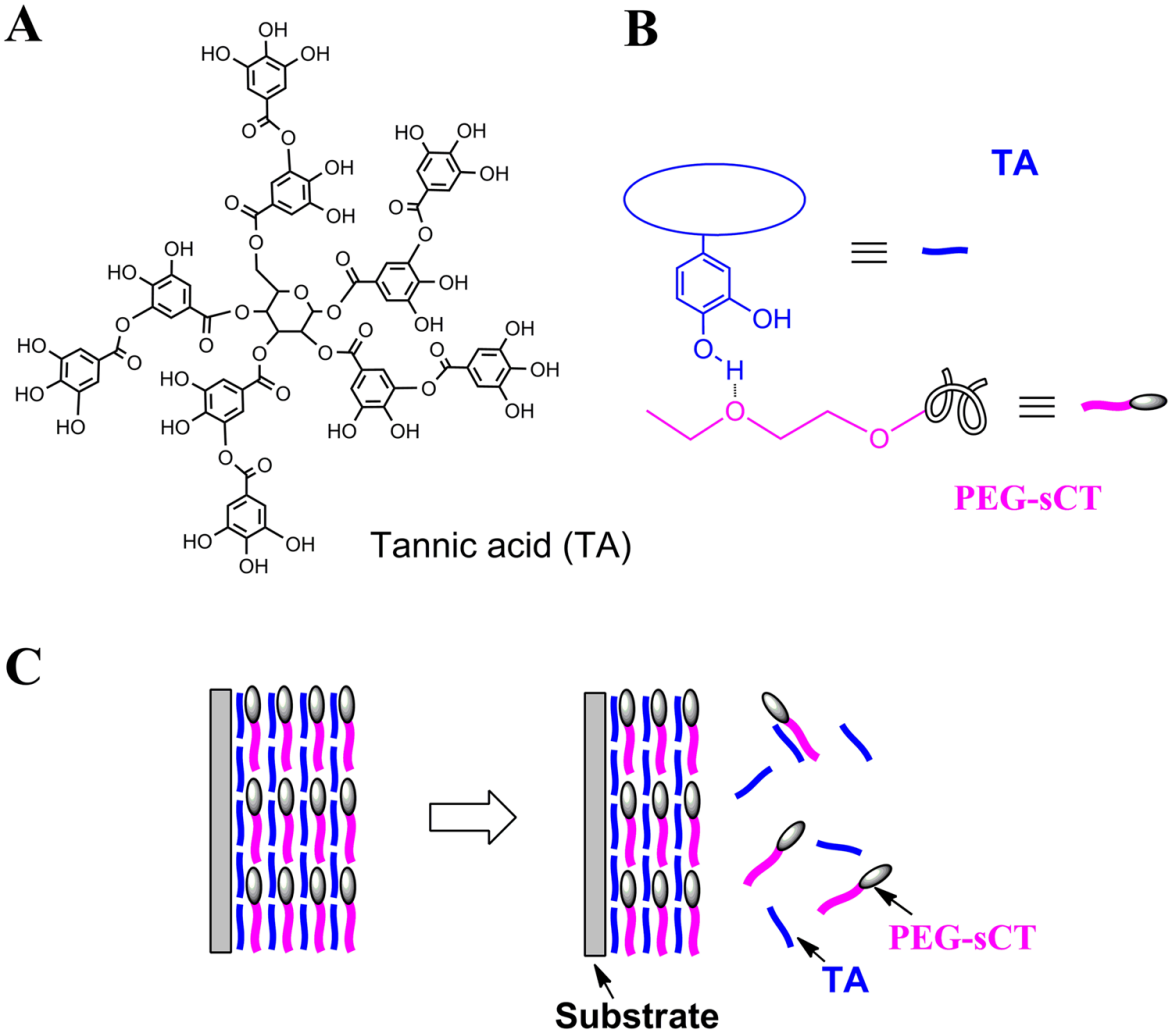
(**A**) Chemical structure of TA. (**B**) Hydrogen bonding between TA and PEG-sCT. (**C**) Release of PEG-sCT from the hydrogen-bonded PEG-sCT/TA film as a result of the gradual disintegration of the film.

**Figure 2:**
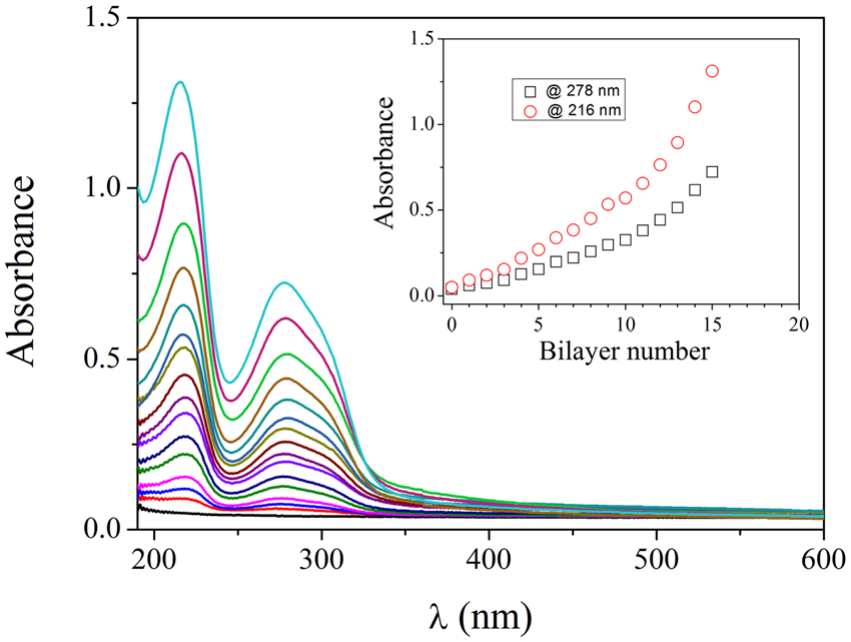
UV-vis absorption spectra of PEG-sCT/TA films with various bilayer numbers (0–15). Inset: Plot of absorbance at 216 and 278 nm against the bilayer number. Both bands can be assigned to phenyl groups in TA.

**Figure 3:**
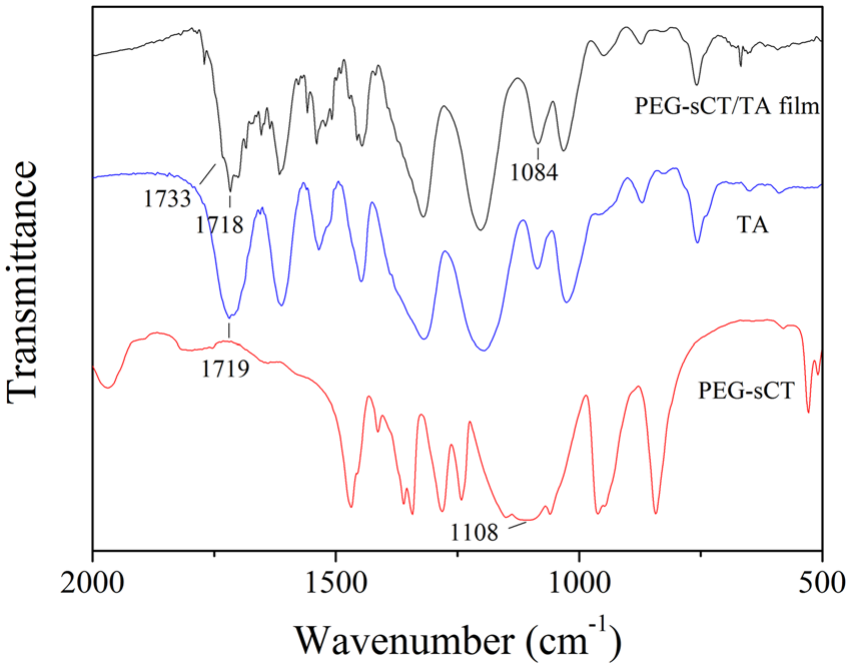
FTIR spectra of PEG-sCT, TA and a PEG-sCT/TA film.

The new carrier designed here is the hydrogen-bonded layer-by-layer (LBL) film of a PEGylated peptide and a polyphenol (Fig. 1 and Figure S1). The peptide drug is first PEGylated and then incorporated into a thin film by layer-by-layer assembly^31^ with a polyphenol. As a proof of concept, PEGylated salmon calcitonin (PEG-sCT)^32^, an attractive therapeutic peptide for the treatment of bone disorders, was chosen as a model peptide drug. Tannic acid (TA), a natural hydrolysable tannin, which itself shows antimutagenic, anticarcinogenic, antimicrobial, antioxidant, and antibacterial activities^33^, was chosen as the polyphenol (Figure 1A). The LBL films were fabricated by dipping quartz slides in aqueous solutions of PEG-sCT and TA alternately. As shown in Fig. 2, the film absorbance increases with increasing dipping cycles, suggesting the successful deposition of PEG-sCT and TA onto the substrate. It is noteworthy that only a small portion of drug in the assembly solution is incorporated into the LBL film. It is expected that loading efficiency can be improved by repeated using of the PEG-sCT and TA solutions. The main driving force for the film buildup is supposed to be the hydrogen bonding between the PEG chain in PEG-sCT and TA (Figure 1B). As an evidence, a shoulder appears at 1733 cm^−1^ in the FTIR spectra of the LBL film, suggesting some of the intra-molecular hydrogen bonds in TA break as a result of the formation of intermolecular hydrogen bonds between PEG-sCT and TA (Figure 3). The morphology of the films was studied using SEM. Like other LBL films, the PEG-sCT/TA films also present a rough surface^34^ (Figure S2A). The thickness of an 8-bilayer film was measured to be ~20 nm.

**Figure 4:**
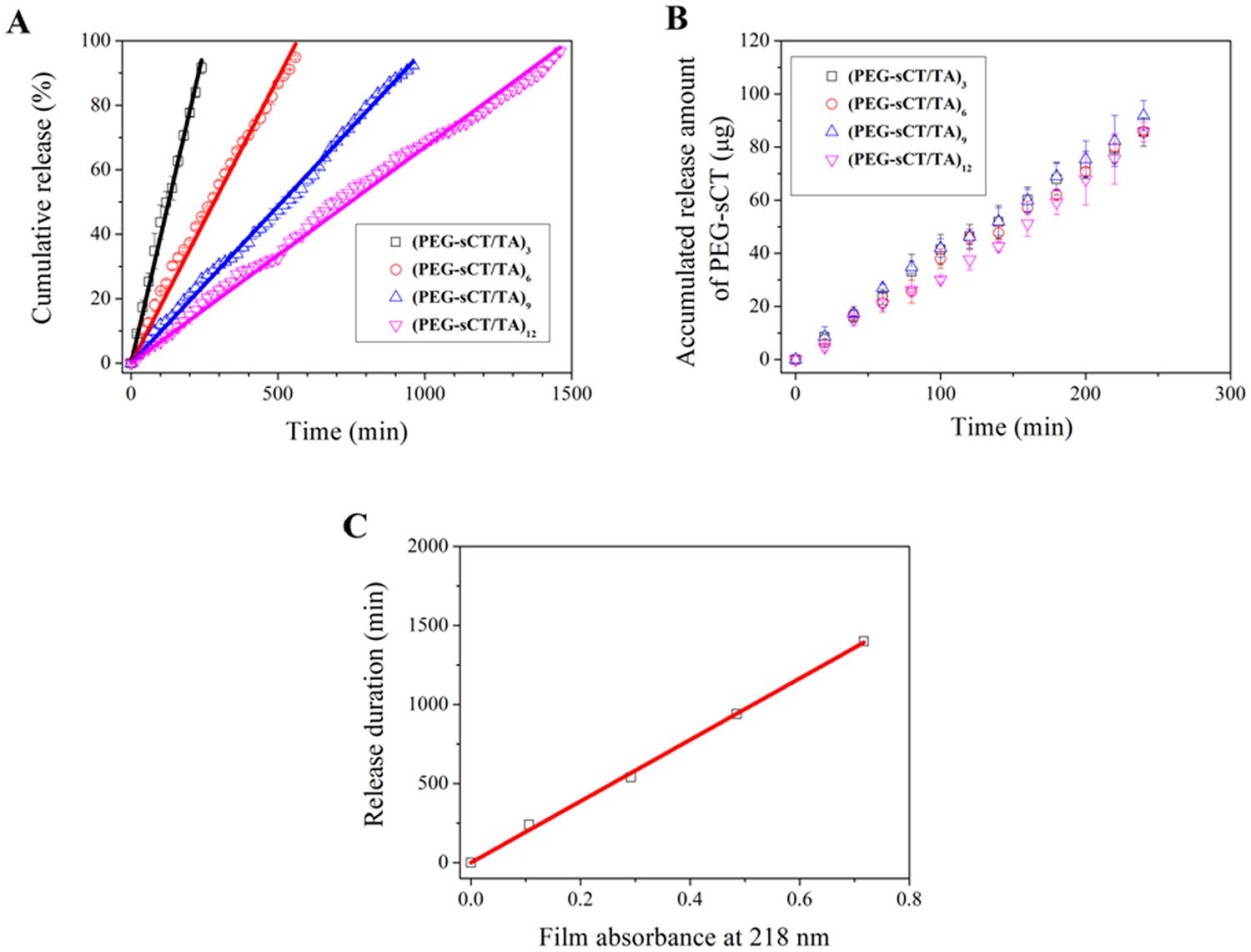
(**A,B**) Release of PEG-sCT from different bilayer number of PEG-sCT/TA films drawn as percentage release (**A**) and cumulative released amount (**B**). (**C**) Release duration (represented as time for 90% release) as a function of film thickness (represented as the absorbance of the original film at 218 nm). Release media: 50 mM pH7.4 phosphate buffer. T = 37 °C.

To study the *in vitro* release of PEG-sCT, the PEG-sCT/TA films were soaked in aqueous solutions and the concentration of PEG-sCT released into the media was analyzed by HPLC (Figure 1C). Figure 4A shows the release profile of PEG-sCT from 4 PEG-sCT/TA films. One can see in all cases the accumulated released amount of PEG-sCT increases linearly with time, with a correlation coefficient >0.99. The result indicates that the films release PEG-sCT at a constant rate. The zero-order kinetics continues even after >90% of PEG-sCT is released. Initial burst release and incomplete release, which are common issues when using biodegradable microparticles^8, 12, 35^ and hydrogels^10^ as peptide-releasing carriers, were not observed here. The morphology of the films does not change during the release (Figure S2).

**Figure 5:**
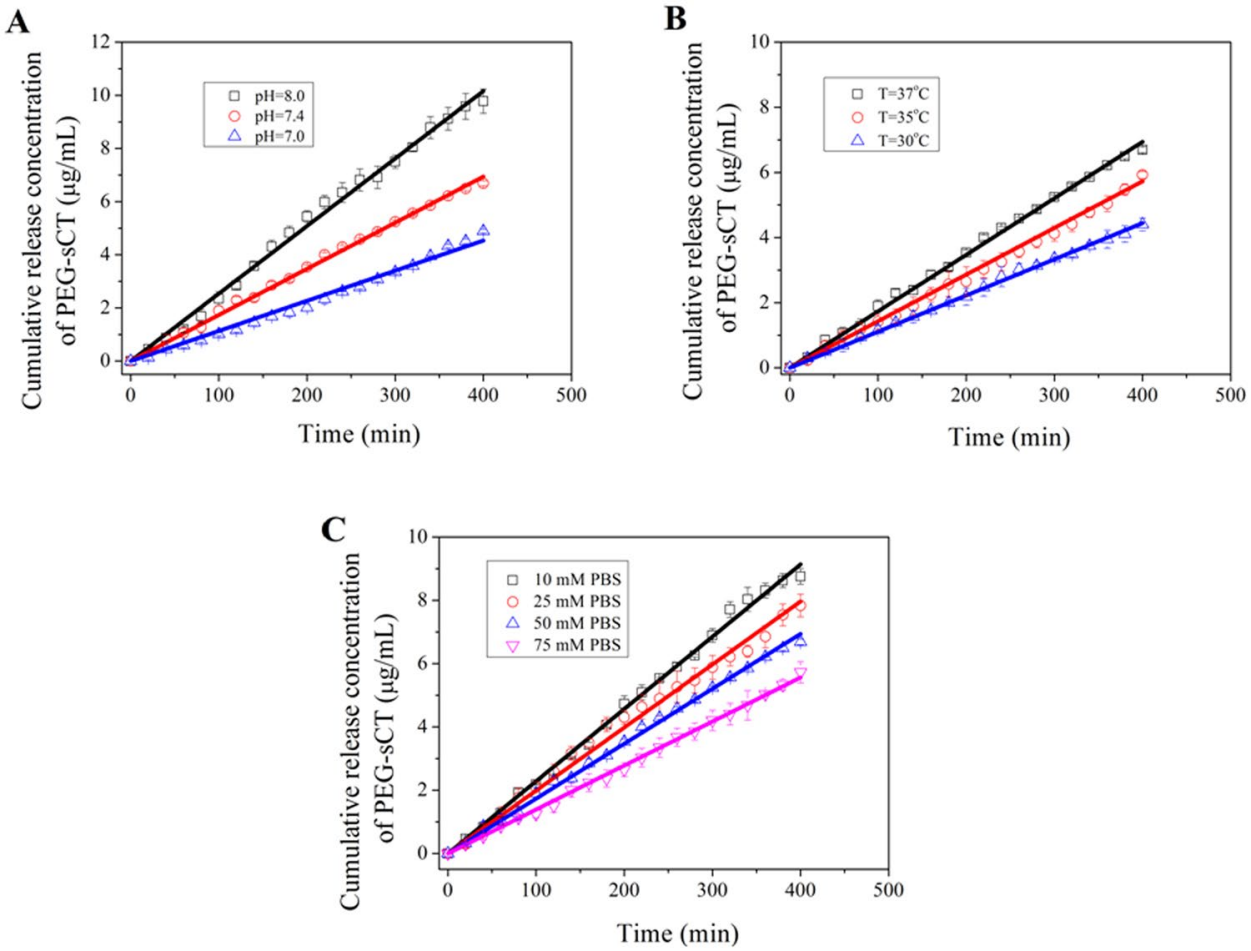
Release profiles of PEG-sCT from (PEG-sCT/TA)_6_ films under various conditions: (**A**) in 50 mM phosphate buffer of various pH as indicated, T = 37 °C; (**B**) in 50 mM pH7.4 phosphate buffer at various temperatures; (**C**) in various concentration of phosphate buffer. pH = 7.4, T = 37 °C.

The effect of a series of factors on the release of PEG-sCT was studied. As shown in Fig. 5A, an increased pH of the release media results in a faster release of PEG-sCT. Higher temperature also accelerates the release of PEG-sCT from the films, (Fig. 5B) however, an increase in the ionic strength of the release media slows down the peptide release (Figure 5C). These results suggest it will be possible to tune the release rate of the peptide via external stimuli. Note although the release rate was changed, the zero-order kinetics was still followed under all these conditions studied.

**Figure 6:**
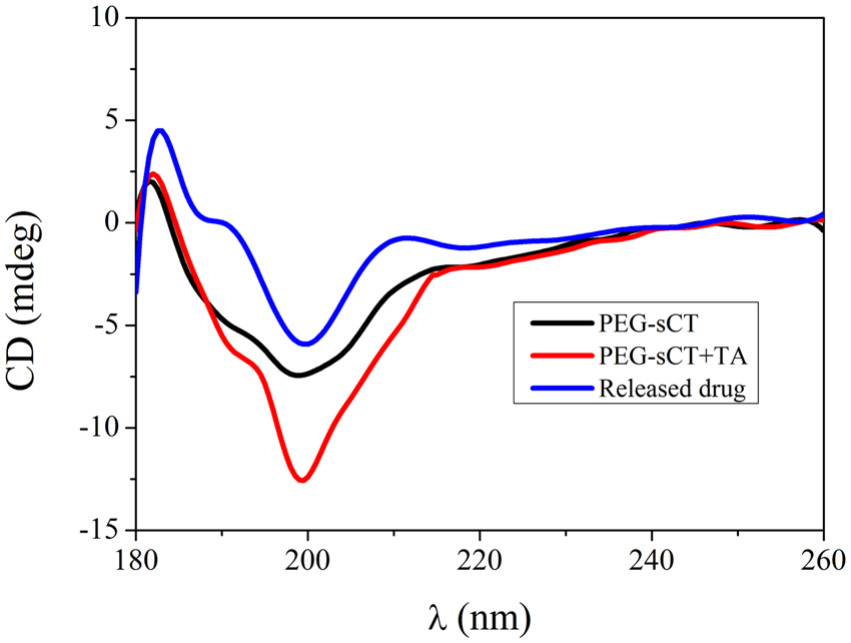
CD spectra of a solution of native PEG-sCT, mixed solution of PEG-sCT and TA, and release media into which PEG-sCT and TA released.

A suitable carrier for peptide should not alter the structure of the incorporated drug. For this purpose, the secondary structure of PEG-sCT released into the media was studied by circular dichroism (CD). As shown in Fig. 6, the native PEG-sCT presents a typical negative band at ~199 nm and a low magnitude of ellipticity at 222 nm, suggesting a major random coil structure with a small helical content^36, 37^. The CD spectra of sCT-PEG released from the film were similar to that of the native drug, suggesting the secondary structure of the peptide remains unchanged.

**Figure 7:**
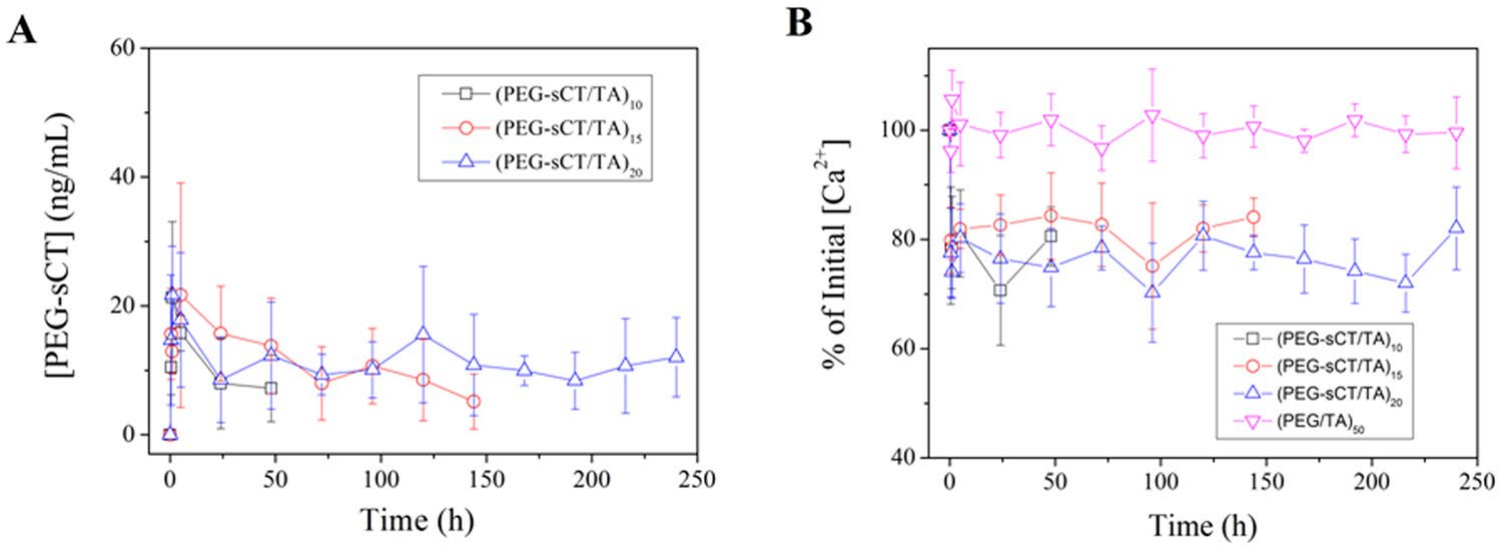
PEG-sCT levels in rat serum (**A**) and plasma calcium levels (**B**) after implantation.

The major biological effect of PEG-sCT is to lower blood Ca^2+^ level^32^. To check if the LBL film could generate a hypocalcaemic response, the films were subcutaneously implanted in SD rats. As shown in Fig. 7A, PEG-sCT was detected in the blood soon after the implantation. Particularly the plasma concentration of PEG-sCT remains almost constant at ~10 ng/mL before a sudden drop to zero. The result strongly suggests that the *in vivo* release of PEG-sCT also follows a zero-order kinetics. The serum calcium level after the film implantation was shown in Fig. 7B. Compared to the group implanted with PEG/TA, a ~20% decrease in serum calcium level was observed for rats implanted with PEG-sCT/TA films, which is typical calcium level reduction achieved with sCT in rats^38^. The result confirms that the PEG-sCT released from the films remain bioactive *in vivo*. In addition, the calcium level maintains constant in the releasing period of the corresponding PEG-sCT/TA film.

## Discussion

Most drug carriers typically release a drug in a fast-then-slow manner. For example, the drug release from a hydrogel carrier usually follows Higuchi’s kinetics, i.e., the release is proportional to the square root of time, because of a diffusion-controlled release^39^. Quite differently, the release of PEG-sCT from the PEG-sCT/TA films follows a perfect zero-order kinetics. No initial burst release was observed (Figure 4). In addition, the zero-order kinetics is followed for the entire release process (Figure 4A and also Figure S3 in supporting information, in which complete release profiles of a 4-bilayer and 8-bilayer film with data beyond 100% release were shown). In contrast, for many zero-order release carriers reported previously, constant release can only be observed in a certain period of the release process^40^.

The zero-order release of PEG-sCT from the PEG-sCT/TA films can be explained by the unique release mechanism of these films. Unlike common drug carriers which release drug via diffusion or polymer degradation^13^, here PEG-sCT was released via the gradual disintegration of the film (Figure 1C). We^34, 41, 42^ previously observed that hydrogen-bonded LBL films disintegrate gradually in aqueous solutions, because the film materials are bonded with dynamic, reversible hydrogen bonds. It is noteworthy the gradual disintegration of hydrogen-bonded films is different from the pH-induced disintegration of the same films. In the latter case, the films disintegrate as a result of the breakage of hydrogen bonds, which is a consequence of the pH-induced dissociation of the hydrogen-donor, for example PAA (poly(acrylic acid)) in PVPON/PAA (PVPON: poly(vinyl pyrrolidone)) films^43^. It occurs only at a pH higher than the pKa of the hydrogen-donor. In addition, it usually occurs instantly. In contrast in the former case, most of the hydrogen bonds in the film remain intact. It occurs even at a pH much lower than the pKa of PAA as in the case of PVPON/PAA film^41^. The gradual disintegration of PEG-sCT/TA film or PEG/TA film in aqueous solutions was monitored using UV-vis, QCM and Fabry-Perot fringes (Figures S4–S6). Gradual disintegration was also observed from LBL films linked with other dynamic bonds^44^, such as phenylboronate ester bonds^45, 46^ and Schiff base bonds^47^.

As shown in Fig. 1C, the film disintegration, i.e., the dissociation of the film material, PEG-sCT/TA complex, results in the release of free PEG-sCT and TA into the media. Meanwhile free PEG-sCT and TA in the media may deposit back onto the film, or form soluble PEG-sCT/TA complex and remain in the solution. The whole process can be described using the following reactions:

1 $${\rm{P}}{\rm{E}}{\rm{G}}{\textstyle \text{-}}{\rm{s}}{\rm{C}}{\rm{T}}/{\rm{T}}{\rm{A}}\,({\rm{f}}{\rm{i}}{\rm{l}}{\rm{m}})\mathop{\mathop{\rightleftharpoons }\limits^{{{\rm{K}}}_{1}}}\limits_{{{\rm{K}}}_{2}}{\rm{P}}{\rm{E}}{\rm{G}}{\textstyle \text{-}}{\rm{s}}{\rm{C}}{\rm{T}}+{\rm{T}}{\rm{A}}\rightleftharpoons {\rm{P}}{\rm{E}}{\rm{G}}{\textstyle \text{-}}{\rm{s}}{\rm{C}}{\rm{T}}/{\rm{T}}{\rm{A}}\,({\rm{s}}{\rm{o}}{\rm{l}}{\rm{u}}{\rm{b}}{\rm{l}}{\rm{e}}\,{\rm{c}}{\rm{o}}{\rm{m}}{\rm{p}}{\rm{l}}{\rm{e}}{\rm{x}})$$

The net rate of the film disintegration, or, the release rate of PEG-sCT, can be written as:

2 $${\rm{R}}={{\rm{R}}}_{{\rm{d}}{\rm{i}}{\rm{s}}{\rm{s}}{\rm{o}}{\rm{c}}{\rm{i}}{\rm{a}}{\rm{t}}{\rm{i}}{\rm{o}}{\rm{n}}}-{{\rm{R}}}_{{\rm{d}}{\rm{e}}{\rm{p}}{\rm{o}}{\rm{s}}{\rm{i}}{\rm{t}}{\rm{i}}{\rm{o}}{\rm{n}}}={{\rm{k}}}_{1}[{\rm{P}}{\rm{E}}{\rm{G}}{\textstyle \text{-}}{\rm{s}}{\rm{C}}{\rm{T}}/{\rm{T}}{\rm{A}}]-{{\rm{K}}}_{2}[{\rm{P}}{\rm{E}}{\rm{G}}{\textstyle \text{-}}{\rm{s}}{\rm{C}}{\rm{T}}][{\rm{T}}{\rm{A}}]$$

where [PEG-sCT/TA] is the concentration of PEG-sCT/TA complex in the film, [PEG-sCT] and [TA] are concentrations of free PEG-sCT and TA in the media, respectively, and k_1_ and k_2_ the rate constants. Under the experimental conditions, [PEG-sCT] and [TA] in the media are very low. Therefore the second term, i.e., the rate of free PEG-sCT and TA to deposit back, is negligible. The release rate of PEG-sCT can be re-written as

3 $${\rm{R}}={{\rm{R}}}_{{\rm{d}}{\rm{i}}{\rm{s}}{\rm{s}}{\rm{o}}{\rm{c}}{\rm{i}}{\rm{a}}{\rm{t}}{\rm{i}}{\rm{o}}{\rm{n}}}={{\rm{k}}}_{1}[{\rm{P}}{\rm{E}}{\rm{G}}{\textstyle \text{-}}{\rm{s}}{\rm{C}}{\rm{T}}/{\rm{T}}{\rm{A}}]$$

Equation 3 indicates that the release of PEG-sCT is determined by the dissociation of PEG-sCT/TA complex. As a solid-like material, concentration of PEG-sCT/TA complex in the film, [PEG-sCT/TA], could be regarded as constant, therefore the release of PEG-sCT is determined only by the rate constant k_1_. For dynamic films from polymers with a wide molecular weight distribution, k_1_ is larger for species with a lower molecular weight. Consequently those films exhibit a “fast-then-slow” release pattern^34, 45–47^. In contrast here both PEG-sCT and TA have a narrow molecular weight distribution, therefore k_1_ for all species can be regarded to be the same^48, 49^. Therefore the PEG-sCT/TA films disintegrate at a constant rate and releases PEG-sCT at a constant rate.

In Fig. 4B, the release profiles of the 4 films were replotted in terms of cumulative released amount of PEG-sCT, instead of percentage release as in Fig. 4A. One can see these films release PEG-sCT at almost the same rate, despite that their thickness is different. Furthermore as shown in Fig. 4C, the duration for a film to continuously release PEG-sCT is proportional to its thickness. This feature makes the new carrier highly predictable. For a film with a known film thickness, one may predict how long the film will continuously release the drug. On the other hand, one could fabricate a film with a particular thickness according to its predetermined release duration.

The new carrier not only allows for zero-order release of PEG-sCT, but tuning the release rate using external stimuli, such as pH and temperature (Fig. 5). The effects of the external stimuli on the release rate of PEG-sCT can also be explained by the unique release mechanism of the new carrier. An elevated pH increases the dissociation of TA and therefore weakens the hydrogen bonding between TA and PEG-sCT. In addition, with an enhanced dissociation, the electrostatic repulsion among the charged TA molecules increases. Both effects are favorable for the film disintegration. Therefore the release rate of PEG-sCT increases with increasing pH as shown in Fig. 5A. The accelerated release of PEG-sCT at higher temperature, as shown in Fig. 5B, could be attributed to the weakened hydrogen bonding between PEG-sCT and TA. It is well-known that hydrogen bonding is sensitive to temperature and heating could partially break the hydrogen-bonds in hydrogen-bonded LBL films^50, 51^. The retarded release of PEG-sCT at an elevated ionic strength, as shown in Fig. 5C, can be explained by the enhanced screening of the electrostatic repulsion among the charged TA molecules at higher ionic strength.

The key goal of a release system is to maintain the plasma drug concentration within the therapeutic window for a prolonged period of time. For zero-order release carriers, as the drug is released at constant rate, it will be possible to maintain the plasma drug concentration constant. This will avoid a plasma drug concentration which is too high to exert toxic effects, or too low to fail to exert therapeutic effects, and therefore lead to the best outcome. As expected, for rats with implanted PEG-sCT/TA films, the plasma concentration of PEG-sCT remains almost constant (Fig. 7A). When a 20 bilayer film was implanted, the plasma PEG-sCT level can be maintained for 10 days, which is much longer than the half-life of PEG-sCT when administered via injection (~83 min)^38^. Along with a constant plasma PEG-sCT level, the serum calcium level remains almost constant until the serum PEG-sCT level drops to zero. The result suggested that almost all drugs exert therapeutic effects because the drug concentration was remained in the therapeutic window during the whole releasing period. This property is highly desirable considering the high synthetic and production costs of a peptide drug^52^. As mentioned above, when using a carrier with a “fast and slow” release pattern, a significant portion of the drug will not exert a therapeutic effect, because the drug concentration becomes lower than the therapeutic level at the later stage.

In conclusion, using PEG-sCT/TA film as an example, we demonstrated that hydrogen-bonded LBL films of PEGylated peptide and polyphenol could be used for the zero-order release of peptide drugs. The films were fabricated under mild conditions without the use of any harmful solvents. The second component, TA, is a natural product and presents potential therapeutic activities itself. The *in vitro* release profile of the peptide suggests a zero-order kinetics without initial burst release. In addition the release rate could be tuned via external stimuli, such as pH and temperature. *In vivo* test showed that the implanted LBL films could maintain the plasma level of PEG-sCT constant over an extended period and thus keep a constant, lowered serum calcium level. Further studies using microspheres as substrate for film fabrication, which will allow administration via subcutaneous injection, are under way.

## Methods

### Materials

Linear salmon calcitonin ((D)Ala-Ser-Asn-Leu-Ser-Thr-Cys-Val-Leu-Gly-Lys-Leu-Ser-Gln-Glu-Leu-His-Lys-Leu-Gln-Thr-Tyr-Pro-Arg-Thr-Asn-Thr-Gly-Ser-(D)Ala-Thr-Pro-NH_2_, sCT) (purity >95%) was purchased from Hangzhou SINOPEP Pharmaceutical Inc. Methoxy poly(ethylene glycol)-maleimide (mPEG-Mal) (M_w_ = 5 K) was purchased from Beijing Kaizheng Biothch Development Co. Ltd. Tannic acid (TA) and poly(ethylene glycol) (PEG, M_w_ = 5 K) were purchased from Sigma-Aldrich. The chemicals were used as received without further purification. Salmon calcitonin ELISA Kit was purchased from Shanghai Enzyme-linked Biotechnology Co., Ltd. Calcium Colorimetric Assay Kit was obtained from Genmed Co., Ltd, Shanghai.

### Synthesis of PEGylated salmon calcitonin (PEG-sCT)

PEG-sCT was synthesized by the reaction between the maleimide group at the end of mPEG-Mal and 7-Cys in sCT. Briefly, 2.0 g of sCT was dissolved in 30 mL of 0.2 M NaH_2_PO_4_ in 50 mL Erlenmeyer flask. Then 4.10 g of mPEG-Mal was added into the solution. The reaction was allowed to proceed for 1 hour. HPLC was used to monitor the reaction until the reaction finished. The crude product was purified by Biotage SP-1 middle-pressure preparative liquid chromatography (MPLC) to >98% purity with RP-C18 column (SepaFlash HP, 40–60 μm (SP), Santai Technologies, Inc.). The product was collected, concentrated and lyophilized. The yield was 3.15 g. The purity of product was determined by analytical HPLC (SHIMADZU SPD-10A) with RP-C4 column (Phenonmenex, Jupiter 5 u, 4.6 × 250 mm, 300 Å) at a flow-rate of 1.0 mL/min (Figure S7A). The molecular weight of the peptides was confirmed by MALDI-TOF mass spectra (Figure S7B).

### Fabrication of LBL films

The PEG-sCT/TA LBL films were fabricated using quartz slides with a size of 44 mm × 10 mm × 1 mm as substrate (Figure S1). Before use the slides were cleaned in boiling piranha solution (3:7 v/v H_2_O_2_−H_2_SO_4_ mixture) (*caution: this solution is extremely corrosive!*), rinsed thoroughly with deionized (DI) water, and then dried. PEG-sCT and TA solutions, both with a concentration of 0.5 mg/mL, were prepared using 10^−3^ M HCl. To fabricate the films, the substrates were immersed in PEG-sCT and TA solutions alternately, each for 5 min, intermediated with twice washing in 10^−3^ M HCl, each for 1 min. This cycle was repeated until the desired bilayer numbers were reached. PEG/TA films were fabricated similarly. The films were not released from the substrate and used as prepared in the following tests.

### ***In Vitro*** Release of PEG-sCT

The LBL films were immersed in 20 mL of release media (pH7.4 50 mM phosphate buffer, if not otherwise specified) which were incubated at 37 °C (if not otherwise specified). A rotary table was used to keep the release media homogeneous. Every 20 minutes the release media was completely withdrawn and replaced with same volume of pre-warmed fresh media. The concentration of peptide in the release media was measured by HPLC. To measure the total amount of peptide in the films, they were completely disintegrated by immersion in pH9.0 50 mM phosphate buffer. All experiments were carried out in triplicate. The results were reported as mean ± standard deviation.

### ***In vivo*** release and hypocalcemic efficacy

*In vivo* release of PEG-sCT was carried out with male Spraguee Dawley (SD) rats (200–240 g). The SD rats were cared in accordance with international standards on animal welfare. The rats were divided into 4 groups (n = 5 for each group). The control group was treated with PEG/TA film, while the other 3 groups were treated with PEG-sCT/TA film with various bilayer numbers. The size of the films was all 10 mm × 10 mm × 1 mm. The rats were first anesthetized with sodium pentobarbital (65 mg/kg i.p.). The film was then implanted subcutaneously at the back of each rat through a small incision in the neck region. An i.v. catheter was inserted into the jugular vein using standard aseptic surgical procedures. Blood samples were collected from the jugular vein at pre-determined time intervals. All samples were centrifuged at 10,000 rpm for 10 min and serum was collected. They were stored at −10 °C before analysis. Concentrations of PEG-sCT and calcium were determined using Salmon calcitonin ELISA kit and Calcium Colorimetric Assay Kit, respectively. All experimental procedures were conducted in accordance with the Guide for the Care and Use of Laboratory Animals of the China National Academy of Sciences, and were approved by the Animal Care and Use Committee of the Beijing Institute Pharmacology & Toxicology.

### Other characterizations

UV-vis absorption spectra were recorded on a UV-1800 spectrophotometer (SHIMADZU, Japan). Fourier transform infrared (FTIR) spectra were measured on Bio-Rad FTS-6000 spectrometer. CD measurement was performed on a Biologic MOS-450 (Claix, France) at room temperature in a 0.1 cm path cell. The CD signals were recorded from 190 to 260 nm using a bandwidth of 4.0 nm and a scanning rate of 50 nm/min. SEM images were recorded on an FEI NanoSEM 430 Scanning Electron Microscope. Atomic force microscopy (AFM) images were acquired using a Benyuan CSPM5000s scanning probe microscope in tapping mode.

### Statistical analysis

All experiments were repeated at least three times. The results were expressed as mean ± SD.

## Electronic supplementary material

Supplementary Information
