# Supplementary material for: A Drug Carrier for Sustained Zero-Order Release of Peptide Therapeutics
Source: Sci Rep. 2017 Jul 17;7:5524. doi: 10.1038/s41598-017-05898-6 (PMC5514143; doi:10.1038/s41598-017-05898-6)
Supplement: Supplementary file 1 — Supplementary Information [file 41598_2017_5898_MOESM1_ESM.doc]

*Supporting information for*

A Drug Carrier for Sustained Zero-Order Release of Peptide Therapeutics

Ya-Nan Zhao, ^1^ Xiaoyu Xu, ^2^ Na Wen,^1^ Rui Song, ^2^ Qingbin Meng,^2^ Ying Guan, ^1^ Siqi Cheng,^2^ Danni Cao,^2^ Yansheng Dong,^2^ Jiankun Qie,^2^ Keliang Liu^2^ &Yongjun Zhang^1^

Figure S1. (A) Fabrication of LBL films of PEG-sCT and TA by dipping the substrate into PEG-sCT and TA solution alternately, intermediated by water washing. (B) When soaking in PEG-sCT solution, a layer of PEG-sCT will be deposited onto the substrate due to hydrogen bonding between PEG-sCT and the substrate (1). The loosely adhered PEG-sCT will be removed by washing in water (2). The substrate will then be soaked in TA solution. Due to hydrogen bonding between PEG-sCT and TA, a layer of TA will be deposited (3). Similarly the loosely adhered TA will be removed by washing in water (4). Repeating the alternate deposition process will result in a layer-by-layer PEG-sCT/TA film.


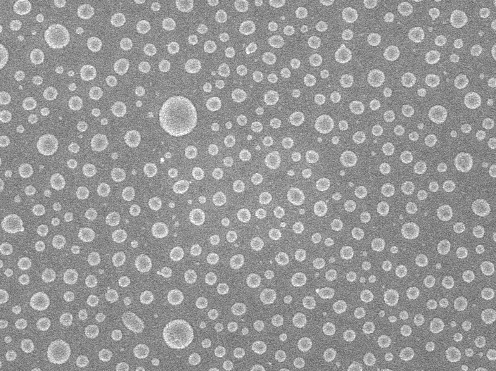

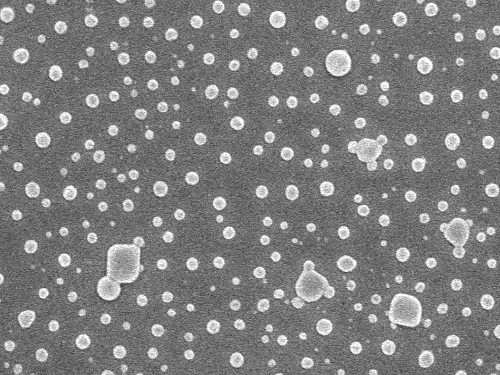


**B**

**A**

**D**


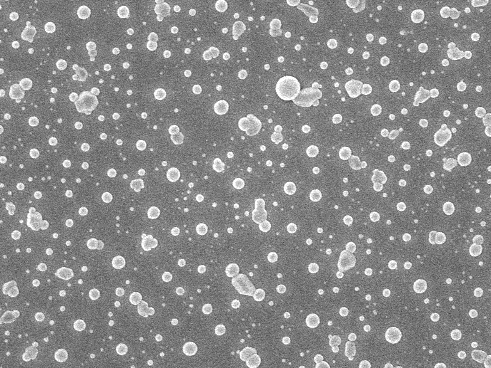

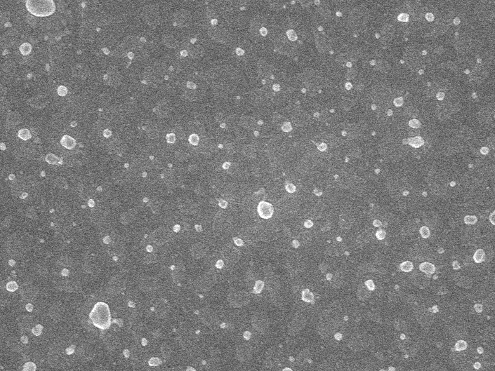


**C**

**E**


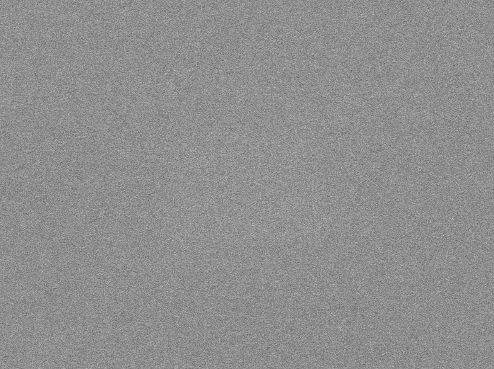


**（F）**

**1 μm**

Figure S2. SEM images of PEG-sCT/TA films. (A) an as-prepared 8-bilayer film. (B-E) 8-bilayer films after in vitro release for 100 min (B), 200 min (C), 400 min (D) and 1000 min. The morphology of the films does not change during the release. 1000 min release results in complete disintegration of the film.

Figure S3. Complete release profiles of PEG-sCT from a 4-bilayer (□) and an 8-bilayer (○) PEG-sCT/TA film. In both cases, the accumulated released amount of PEG-sCT increases linearly with time. A plateau appears at 100% release.

Figure S4. Tracing the gradual disintegration of a 10 bialyer PEG-sCT/TA film in 50 mM pH7.4 phosphate buffer at 37^o^C by UV-vis spectra. The film was fabricated on quartz slide. It was soaked in the buffer, and the media were changed regularly. At proper intervals, the absorbance of the film was measured. The gradual decrease of the absorbance of the film at 218 nm with increasing soaking time suggests the gradual disintegration of the film.

Figure S5. QCM monitoring the disintegration of PEG-sCT/TA film in 50 mM pH8.0 phosphate buffer. The ﬁlm was assembled off-line on the substrate. It was originally soaked in DI water. At proper intervals, phosphate buffer was injected into the flow cell at a steady fast speed, as indicated by the arrows, to replace the solution in the chamber. The following measurement was then conducted under static conditions. The operation was repeated 10 times. Each time an increase in the oscillation frequency was observed, indicating mass loss of the ﬁlm. The device used is a Quartz Crystal Microbalance (QCM200) from Stanford Research Systems, Inc. (USA) The measurement chamber formed by the Teflon holder and flow cell was about 150 μL. (Ref: Liu, S.; Zhou, D.; Guo, T., Biosensors and Bioelectronics, 2013, 42, 80-86; Zhu, Y.; Tong, W. J.; Gao, C. Y., Soft Matter, 2011, 7(12), 5805-5815)

Figure S6. In situ monitoring of the disintegration of a PEG/TA film using Fabry-Perot fringes in its reflection spectra. (A) Reflection spectra of a 30 bilayer TA/PEG film immersed in 20 mM pH8.5 phosphate buffer at 25^o^C. Immersion time is 0, 2, 4, 6, 8, 10, 12, 14, 16, 18, 20, 22, 24, 26, 28, 30 min, respectively. The plots are shifted along the vertical axis for clarity. Mw of PEG is 8,000. (B) Optical path length (OPL= n_e_·θ, where n_e_ is the refractive index and θ the film thickness) of the film as a function of immersion time. OPL was calculated from the two adjacent wavelengths, λ_p_ and λ_p+1_, for which the absorbance is maximal, using the following function: $OPL=n_{e}\theta=\frac{1}{2\left(1/\lambda_{p}-1/\lambda_{p+1}\right)}$. The result indicates that the OPL of the film (and hence the thickness of the film) decreases gradually with time when immersed in the buffer. (Ref: Guan, Y., et al., Journal of Physical Chemistry B, 2006. 110(27): p. 13484-13490; Zhang, X., Y. Guan and Y. Zhang, Biomacromolecules, 2012. 13(1): p. 92–97.)

Figure S7. RP- HPLC proﬁle (A) and MALDI-TOF mass spectra (B) of puriﬁed PEG-sCT.
